# Methodological Approaches To Assess Innate Immunity and Innate Memory in Marine Invertebrates and Humans

**DOI:** 10.3389/ftox.2022.842469

**Published:** 2022-02-18

**Authors:** Manon Auguste, Daniela Melillo, Annunziata Corteggio, Rita Marino, Laura Canesi, Annalisa Pinsino, Paola Italiani, Diana Boraschi

**Affiliations:** ^1^ Department of Earth, Environment and Life Sciences, University of Genova, Genova, Italy; ^2^ Institute of Biochemistry and Cell Biology (IBBC), National Research Council (CNR), Napoli, Italy; ^3^ Stazione Zoologica Anton Dohrn, Napoli, Italy; ^4^ Institute of Translational Pharmacology (IFT), CNR, Palermo, Italy; ^5^ Shenzhen Institute of Advanced Technology (SIAT), Chinese Academy of Science (CAS), Shenzhen, China

**Keywords:** innate immunity, innate memory, invertebrates, humans, *in vivo*, *in vitro*

## Abstract

Assessing the impact of drugs and contaminants on immune responses requires methodological approaches able to represent real-life conditions and predict long-term effects. Innate immunity/inflammation is the evolutionarily most widespread and conserved defensive mechanism in living organisms, and therefore we will focus here on immunotoxicological methods that specifically target such processes. By exploiting the conserved mechanisms of innate immunity, we have examined the most representative immunotoxicity methodological approaches across living species, to identify common features and human proxy models/assays. Three marine invertebrate organisms are examined in comparison with humans, *i.e.*, bivalve molluscs, tunicates and sea urchins. *In vivo* and *in vitro* approaches are compared, highlighting common mechanisms and species-specific endpoints, to be applied in predictive human and environmental immunotoxicity assessment. Emphasis is given to the 3R principle of Replacement, Refinement and Reduction of Animals in Research and to the application of the ARRIVE guidelines on reporting animal research, in order to strengthen the quality and usability of immunotoxicology research data.

## Introduction

Innate immunity is the immune defensive system displayed by living organisms, from plants to mammals. For over 99% of living species, innate immunity is the only immune defensive system, while the most recent adaptive immunity has developed in higher vertebrates and is active in parallel with innate immunity (Gourbal et al., 2018). Inflammatory reactions are innate immune responses aiming at defending the organism from pathogens or diseases, which are tightly regulated in order to achieve pathogen elimination without causing collateral damage to affected tissues/organs. However, in some circumstances, inflammation may lose its controlling mechanisms, thereby causing damage and pathologies (e.g., chronic inflammatory diseases). To examine how innate immunity reacts to foreign substances, toxicants or contaminating particles, it is necessary to adopt immunotoxicological methods that allow us to discriminate between a physiological defensive reaction and an uncontrolled pre-pathological response (Villeneuve et al., 2018). In this context, the use of invertebrate models that display many conserved innate immune mechanisms and are suitable for *in vivo* testing offers excellent possibilities for realistic and meaningful immunotoxicological assessments for every kind of toxicant, both chemical and particulate.

In addition to human models, here we will address three marine invertebrates with different characteristics, the mollusc *Mytilus galloprovincialis* (Mediterranean mussel), the echinoderm *Paracentrotus lividus* (Mediterranean sea urchin) and the tunicate *Ciona robusta* (sea squirt). These three invertebrates were chosen for some specific advantages in toxicological studies, as detailed below. They all share a number of conserved innate immune functions with human beings, which make them suited as human proxys relative to those functions, in addition to representing models for environmental toxicology. The advantages and disadvantages of these four models for immunotoxicological assessment are summarised in Table 1.

**TABLE 1:** SWOT analysis of animal species for immunotoxicological assessment.

Species	Strengths	Weaknesses	Opportunities	Threats
Mussel	• Knowledge on anatomy and physiology	• No long-term cell cultures/cell lines	• Non-invasive sampling immune cells	• Seasonal variability (due to environmental factors, *e.g.*, temperature, presence of natural pathogens)
	• Cosmopolitan, allow comparison	• Bias on cell subtypes (granulocytes more represented), except in cytofluorimetry	• No ethical problems	
	• Simple, easy to handle	• Inter-individual variability (high intraspecific genome and environment-related diversity)	• Aquaculture species, not endangered	
	• Suitable for *in vitro* and *in vivo* experiments		• Resilient to biotic and abiotic stressors	
Tunicates	• Cosmopolitan invasive species	• No long-term cell cultures/cell lines	• Non-invasive haemolymph withdrawal	• Poor molecular characterization of haemocytes/immune cells
	• Simple, easy to handle	• Many haemocyte types with different activities	• No ethical problems	• Seasonal variability
	• Suitable for *in vitro* and *in vivo* experiments	• Inter-individual variability if coming from different environments		• Non-edible species
	• Colonial species suitable for allorecognition studies			
Sea urchin	• Low maintenance and handling costs	• Maintenance in aquaria without stress is challenging	• Medium-term primary cell culture	• Rare in some coastal environments
	• Knowledge on anatomy and physiology	• Very high number of immune receptor-encoding genes	• No ethical issues	• Seasonal variability
	• Versatility (enough to satisfy *in vitro* and *in vivo* experimental needs)	• Stable cell lines unavailable	• Resilience and adaptation	
		• Unknown variability between different environments	• Longevity and lack of neoplastic diseases	
			• Close genetic relationship with humans	
Human (*in vitro*)	• Validated workflows and methodologies	• Mainly *in vitro* or *ex-vivo* experiments possible	• Non-invasive sampling of immune cells	• Inter- and intra-laboratory reproducibility of methods and reagents
	• Possibility to reproducing *in vitro* the human physiological responses	• Ethical approval required	• Development of high-throughput methodologies	• Inter-donor variability (*e.g.*, genetics, immunobiography)
	• Availability of long-term cell culture (including cell lines)		• Development of a Human Screening Platform	
	• Availability of many well characterized reagents (*e.g.*, monoclonal antibodies)		• Higher predictivity of human cell-based models vs. other animal models	

Bivalve molluscs (*i.e.*, oysters, mussels, clams) are widespread in all aquatic environments. Due to their sessile, suspension-feeding habits, they can accumulate a wide range of contaminants and microorganisms from the surrounding medium. Among bivalves, marine mussels (*Mytilus* spp.) are worldwide used as model organisms in biomonitoring environmental quality, as well as to investigate the responses to anthropogenic stressors and potential pathogens (Beyer et al., 2017; Canesi et al., 2017).

Tunicates are marine filter-feeding invertebrates classified within the phylum *Chordata*, which includes all animals with dorsal nerve cords and notochords (as human beings). The largest class in tunicates is represented by ascidians, a cosmopolitan group with a sessile lifestyle (living attached to docks, rocks or boats). Since this exposes them to chronic environmental pollution, they represent a good model to study the effect of contaminants on their immune responses (Taketa and De Tomaso, 2015; Holland, 2016).

Echinoderms are widely distributed across all seas, climates, and marine benthic environments, and include filter feeders, predators, scavengers, grazers and omnivores. The sea urchin *Paracentrotus lividus* is constantly exposed to environmental and anthropogenic pressure, including predation, climate changes, pathogens, pollutants (*e.g.*, chemicals, nanomaterials, plastics), which favoured phenotypic plasticity (Smith et al., 2018). The recent annotation of its genome has opened to the use of the sea urchin as a human proxy non-mammalian model (Rast et al., 2006) for *in vitro* and *in vivo* environmental immunotoxicology.

All these invertebrate models have the advantage of complying with the 3R principle of reducing, refining, and replacing animal experimentation, since the use of invertebrates is not considered as animal experimentation. In this perspective, adherence to the ARRIVE guidelines is strongly recommended in order to strengthen the quality and utility of immunotoxicology research. These guidelines have been developed to improve the quality of reporting in research using animals (Kilkenny et al., 2010) and imply that all investigations on animals should describe essential information, including the number and specific characteristics of animals used, details of housing and husbandry, and the experimental, statistical, and analytical methods applied in the study.

Human beings (*Homo sapiens*) are widespread around the globe. The migratory history of human beings, with consequent exposure to different environments, has shaped the evolution of the human immune system for adapting to the variety of parasites/pathogens present in the different environments. Thus, the human immune system is more complex than in invertebrates and, in addition to the conserved innate immunity present in other living organisms, it includes an adaptive immune system, which develops its specific defensive functions upon exposure to pathogens. The human immune functions are modulated by both genetic and environmental factors, and therefore human immune responses are highly variable between individuals (Brodin and Davis, 2017), but also within a single individual over time and history of exposures (“immunobiography”; Grignolio et al., 2014; Franceschi et al., 2017).

## Models and Methods for Assessing Innate Immune and Innate Memory Responses

Hereafter, we will describe, in the various animal models, *in vivo* and *in vitro* immunotoxicological approaches, with the indication of the methods/target functions addressed and the assay procedures applied for such assessment (Table 2 and Table 3).

**TABLE 2:** SWOT analysis of *in vivo* animal models for immunotoxicity assessment.

Species	Strengths	Weaknesses	Opportunities	Threats
Mussel	• Large source of cells	• High number of animals needed	• Different routes of exposure (water, dietary, injection)	• Seasonal changes
	• Consider the animal in its whole (circulating fluid and different tissues)	• Long experimental preparations	• Control all external factors	• Dietary exposure (natural) needs accurate quantitative assessment
	• Suitable for studying longer term effects			
	• Possibility to study immune cell metabolism			
Tunicates	• Whole organism/cell population evaluation	• Lack of immune “healthy state” benchmarks	• Use of housed animals or reared-in-house animals to control environmental conditions	• High variability among individuals
	• Identification of tissue-specific responses	• Exposure time is critical	• Examining the role of immunosenescence	• Influence of non-immune parameters on immune functions (age, nutrition, etc.)
	• Possibility to study immune cell metabolism	• Specific functions (*e.g.*, phagocytic rate, degranulation) can only be tested on some cell populations		• Seasonal variability
Sea urchin	• High number of circulating cells	• High number of specimens required	• Possibility of controlling the immunological state (quiescence *vs*. activation)	• Gender differences in immune response
	• Long and repeated experimental exposure possible	• High requirement of space/number of tanks for exposure experiments	• Possibility of a comprehensive understanding of immunity (immune cells, microbiota)	• High baseline immune activity in freshly caught animals (acclimation period necessary)
	• Immune functions not affected by age			• Seasonal variability
	• Possibility to study immune cell metabolism			

**TABLE 3:** SWOT analysis of *in vitro* experimental methods in different animal models for immunotoxicity assessment.

Species	Method/parameter	Strengths	Weaknesses	Opportunities	Threats
Mussel	Analysis of haemolymph and haemocytes	• Study of hemocyte monolayers alone or in whole hemolymph	• Short term cell survival once extracted from the animal (up to 24 h)	• Low number of animals needed	• No cell line available or long-term cultures for routine check
		• Large hemolymph quantity (ml)	• Toxicokinetic studies required for correctly assessing exposure and uptake	• Haemocytes can be kept under non strictly sterile conditions	
		• Can be maintained in easy, cheap medium			
Tunicates	Analysis of haemolymph parameters	•Identification of immunologically active molecules	• Dose- and exposure time-dependent response	• Correlation between humoral factors and haemocyte population composition	• High variability among individuals
			• Toxicokinetic studies required for correctly assessing exposure and uptake		
	RNA expression	• Suitable for tissues and isolated cells	• Lack of immune “healthy state” benchmarks	• Providing a wide immune gene expression profiling in healthy and frail conditions (*e.g.*, age, stress)	• High variability among individuals
			• Need of functional counterpart		
	Haemocytes in suspension	• Automated reading	• Lack of cell-specific markers	• Evaluation of haematopoiesis rate	• Does not reflect the immune functions/changes at the level of the entire organism
		• Functional evaluation			
		• Evaluation of whole haemocyte population *vs*. isolated subpopulations			
	Haemocyte short-term cultures (on plate/slide)	• Easy to manage	• Largely limited to microscopical evaluation (light and fluorescence microscopy)	• Development of assays	• Inter-operator variability
		• Inexpensive	• Few soluble molecules characterized		
		• Fast	• Lack of cell-specific markers		
		• Functional assays possible			
		• RNA expression and protein production possible			
Sea Urchin	Primary cell culture	• Simple optimised protocol	• Limited immune cell proliferation	• Development of assays	• Need of validation of assay predictivity
		• Easy and cheap medium (blood fluid, anticoagulant solution, artificial seawater)	• Limited duration in culture (2 weeks)		
		• Low number of donors necessary	• Skill required in blood withdrawal		
		• High number of cells per donor	• Specific cell density conditions necessary		
		• Mixed cell population or single cell type culture	• Toxicokinetic studies required for correctly assessing exposure and uptake		
Human	Conventional 2D cultures of established monocyte-like cell lines (THP-1, U937)	• Relatively low cost	• Cells are not normal and may not retain the original characteristics and functions	• Possibility of developing excellent, fast and reproducible assays based on valid representative cell functions	• Inter- and intra-laboratory genotypic variations may generate irreproducible and conflicting results
		• Unlimited source of cells	• Use of mouse cell lines for human toxicity testing adds representativity problems		• Risk of examining effects that never occur in real life
		• Limited variations in cell responses	• No microenvironmental and intercellular mechanical and chemical cross-talk		
		• Standardised culture conditions	• Toxicokinetic studies required for correctly assessing exposure and uptake		
	Conventional 2D cultures of primary immune cells derived from donors	• High physiological relevance	• Relatively high cost	• Possibility to detect disease-caused changes in primary immune cell functional phenotypes	• Need of kinetic analysis for examining medium/long-term effects, in most cases impossible because of the short cellular lifespan
		• Possibility to develop tissue-like mature cells (*e.g.*, monocyte-derived macrophages or DC, CD34^+^-derived mast cells)	• Donor-to donor variation (genetic heterogeneity and immunobiography)		
			• More complex culture conditions		
			• Need of technical skills		
			• Low number of cells for functional experiments		
			• Limited microenvironment and intercellular communication		
			• Only short-term cultures possible without substantial alterations of cell functions (*e.g.,* senescence)		
			• Toxicokinetic studies required for correctly assessing exposure and uptake		
	3D co-culture systems	• More accurate representation of *in vivo* scenarios	• Complex culture conditions	• Possibility to evaluate global effects on different cell types	• It cannot reproduce the complexity of architectural microenvironment
		• Reproduction of intercellular communication	• Difficulties for long-term culture of primary cell		• Problem of compatibility between the different cell types and unwanted immune activation
		• Increased relevant cell-to-cell and cell-to-ECM signalling	• Variability of biological matrices may lead to irreproducible results		
			• Toxicokinetic studies required for correctly assessing exposure and uptake		
	Organs-on-chip	• High physiological relevance	• High cost	• Possibility to develop a testing platform for toxicological safety assessment	• Challenging model validation
		• Reproduction of tissue/organ level organization	• Need of specific expertise	• Possibility to integrate cell technology, microenvironment and personalised parameters for the development of precision medicine	• Problem of compatibility between the different cell types and unwanted immune activation
		• Patient-derived models	• Oversimplified organ model		
		• Reproduction of intercellular communication	• Difficult to standardise		
		• Realistically monitoring of human immune cell reactivities	• Toxicokinetic studies required for correctly assessing exposure and uptake		

Special emphasis will be dedicated to innate memory, a defensive immune mechanism that still has not been sufficiently addressed in immunotoxicity assessments, despite its importance in immune defence. Invertebrates are widespread in all environments (terrestrial, freshwater, marine) and face all sorts of natural or anthropogenic stressors, including extensive attacks of pathogens. Their capacity to survive in these habitats depends, in addition to other non-immunological factors, on the efficacy of their immune defensive system (defined as innate immunity). It is evident that innate immunity does not exclusively encompass primary “innate” reactivity, but it could retain a memory of past challenges that generates more effective reactions to chronic or repeated exposure to challenges, a mechanism known as innate immune memory (Milutinovic and Kurtz, 2016; Melillo et al., 2018). In invertebrates, innate memory includes three types of secondary/memory response: 1) recall response, *i.e.*, a higher or more efficient secondary response; 2) sustained unique response or acquired resistance, *i.e.*, a long-term immune activation that can be further upregulated upon secondary exposure; and 3) immune shift, *i.e.*, the upregulation of another, more efficient immune mechanism upon subsequent exposures (Milutinovic and Kurtz, 2016; Melillo et al., 2018). Innate memory is also maintained in human beings, in parallel to adaptive memory. The innate immune memory in humans essentially corresponds to the recall response of invertebrates, but it can go in two directions, *i.e.*, a higher response (potentiation, trained immunity) or tolerance (Foster et al., 2007; Ifrim et al., 2014; Netea et al., 2020; Divangahi et al., 2021).

Many seasonal factors, including temperature and salinity fluctuation, food availability, and reproductive period, can lead to substantial changes in the innate immune parameters, thereby influencing the experimental design for immunotoxicological testing (Hutchinson and Manning, 1996; Carver et al., 2006; Donaghy and Volety, 2011). The role of environmental factors in modulating immune responses is recognized as an integral part of marine life and should be always considered. Thus, environmental conditions need standardization as part of the experimental design, in order to develop robust and reproducible immunotoxicological protocols in marine animal models.

### Mussels

#### Immunity and Immune Response Components

The main compartment of interest for studying innate immune responses in bivalves is the circulating fluid, the haemolymph, and is in direct contact with tissues, since the circulatory system is open. Bivalve haemolymph contains circulating cells, the haemocytes (granulocytes and agranulocytes), whereas its soluble component (serum) carries a large range of humoral defence factors (*e.g.*, lectins, hydrolytic enzymes, antimicrobial peptides). All these components interact in providing a coordinated innate immune response. Mussel haemocytes encompass several subpopulations, representing the progressive maturation stages of a single cell type, in particular R1 (large granular cells), R2 (small semi-granular cells) and R3 (small agranular or hyaline cells), involved in different immune defence mechanisms (García-García et al., 2008). The different cell types can show distinct sensitivity/reactivity to different stimuli. Whatever the exposure conditions, after immune challenge the whole haemolymph can be withdrawn from the posterior adductor muscle sinus *via* a non-invasive method. Besides haemolymph, immune responses and inflammatory reactions can take place in other tissues depending on the nature of the contaminant or pathogen. The tissues mostly involved in immune reactions are the gills and digestive gland, due to their roles in filtration, feeding, and intracellular digestion.

#### Routes of Exposure

The immunotoxicity in mussels can be evaluated in the laboratory, either considering the whole animal through *in vivo* exposure or isolated haemocytes for *in vitro* testing.

**FIGURE 1:**
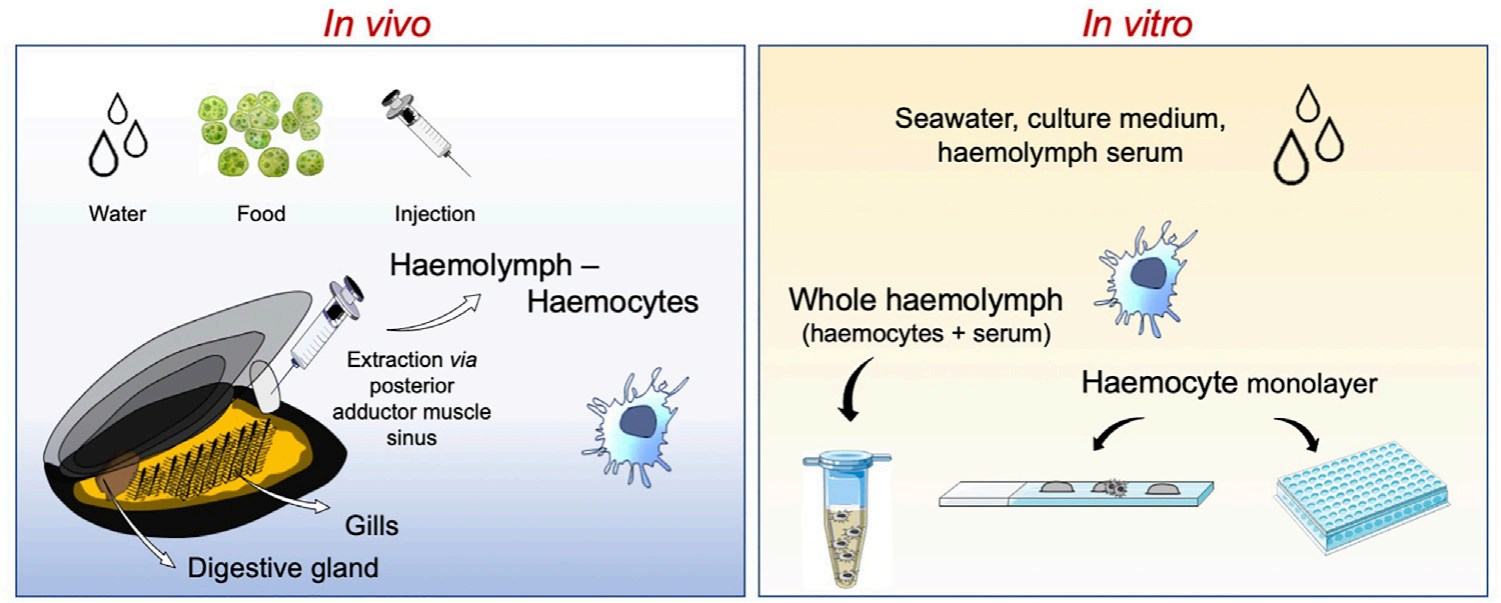
Schematic representation of experimental immune assessments using the mussel *Mytilus galloprovincialis.* Studies can be performed at the whole animal level with *in vivo* experiments or on isolated haemocytes and haemolymph with *in vitro* assays.

Multiple routes of exposure can be used in *in vivo* laboratory experiments, the most common being through the water (Figure 1). This mode of exposure resembles the natural conditions and allows for controlling the concentrations of each stimulus (chemicals or micro-organisms) for each individual (Canesi et al., 2014; Capolupo et al., 2021). Exposure *via* a dietary route integrates complementary concepts, as both contaminants and micro-organisms can be associated with food (*i.e.*, microalgae, particulate matter in suspension), although there is not clear evidence about the exact amount transferred to the animal (Duroudier et al., 2019). Finally, to ensure the direct contact with immune cells, potential chemical or biological immune modulators are directly injected *via* the sinus present in the posterior adductor muscle (Balbi et al., 2019; Rey-Campos et al., 2019).

For *in vitro* testing, *Mytilus* haemocytes represent a suitable tool for immune evaluation (due to their high antibacterial activity, haemocytes can be kept under non strictly sterile conditions). The main limitation of *in vitro* experiments with mussel haemocytes is represented by the need of using primary cell cultures, since no stable long-term cultures or cell lines have been established so far (Canesi et al., 2017). Therefore, assays are carried out on primary haemocyte cultures maintained for very short periods (up to few hours) in sterile artificial seawater or natural medium (haemolymph soluble fraction or serum) or over 24 h in synthetic basal media adjusted for ionic strength (Katsumiti et al., 2015; Auguste et al., 2018). This is however only a partial limitation, because mussel haemocytes have the characteristic of building rapid functional responses, which can be observed within 1 h from exposure. Assays can be carried out on haemocytes in suspension or in monolayers, taking advantage of the capacity of these cells to easily adhere to virtually any surface (such as plastic or glass). Adhesion capacity is particularly strong for granular haemocytes, which are the majority of circulating cells.

#### Systemic Immune Responses

A range of immunological biomarkers has been identified, which provide a quantitative evaluation of the immune responses to chemicals and micro-organisms. These biomarkers can be evaluated at different levels of biological organization (from molecular to organism level).

General haemocyte parameters (cell viability or apoptosis) can be estimated first to get an overview of the effects caused by different contaminants, but alone do not provide details on specific immune-related mechanisms of action. In the circulating fluid, quantitative analysis of the total haemocyte count (THC), and in particular the percentage of the different subpopulations can allow identifying specific activities induced by different stimuli. Flow cytometric techniques, which can evaluate cell size and complexity, are robust and sensitive methods for quantifying these parameters (Renwrantz et al., 2013; Sendra et al., 2020).

The most common targets for immunotoxicity assessment in haemocytes are changes in the lysosomal compartment (such as membrane disruption, change in pH, and changes in number), effects on phagocytosis (index, rate), oxidative burst (release of reactive oxygen species -ROS- or nitric oxide -NO-), and production of lytic enzymes (*e.g.*, lysozyme). Other parameters can be evaluated in the nucleus, such as DNA damage to assess the genotoxicity of some contaminants. A combination of microscopic and spectrophotometric techniques can be used to appraise these parameters (detailed in the section on assays).

Mussel immune cells express a wide range of inducible immune-related genes encoding extracellular recognition and effector proteins, including lectins, peptidoglycan recognition proteins, lipopolysaccharide (LPS) and β1,3-glucan-binding proteins, and antimicrobial peptides. In the absence of functional tests, the transcription of immune-related genes can be assessed by qRT-PCR (Auguste et al., 2020).

Several immune-related markers can be monitored in gills, a complex organ involved in respiration and feeding, and therefore in direct contact with the surrounding medium. In whole gill samples, microscopical techniques can reveal several responses (*i.e.*, enlargement of gill lamellae, increase in infiltrating haemocytes, uptake of foreign objects or pathogens) (Katsumiti et al., 2015; Balbi et al., 2017). Similar to haemocytes, DNA damage or production of NO and ROS by gills can be monitored (Canesi et al., 2014; Balbi et al., 2017). In addition, transcriptomic analysis can show the activation of immune effector immune mechanisms in gills in response to bacterial infection (Saco et al., 2020).

A very important immune compartment is mucosal immunity. In bivalves, the mucus is produced by different epithelia, including the gills, and is involved in particle capture for both feeding and first defence against foreign material. Mucus not only is an excellent physical barrier, but it also contains hydrolytic enzymes, such as lysozyme or proteases, thus representing an efficient defensive system that traps and destroys/neutralizes potential pathogens before they enter the body (reviewed in Allam and Pales Espinosa (2016). Although these studies highlight the diversity of immune effectors based on mucosal proteomics, standardized methods are not yet available to assess mucosal immunity in bivalves.

Other tissues are involved in systemic immune responses, in particular the hepatopancreas or digestive gland. Immune responses in these tissues are generally evaluated by transcriptomics (reviewed in Balbi et al., 2021) rather than using specific functional immune assays.

#### Immune Memory

Studies in bivalve molluscs on secondary immune response after *in vivo* challenge with a pathogen showed enhanced phagocytic capacity and higher bactericidal activity (Cong et al., 2008; Ciacci et al., 2009a; Zhang et al., 2014). It was only recently that the above-mentioned effects were attributed to innate memory-like processes, thereby fostering newly designed experimental approaches/protocols (Lanz-Mendoza and Contreras-Garduño, 2021). In bivalves, most studies investigated the responses to natural pathogens (Rey-Campos et al., 2019; Lafont et al., 2020), but evidence is also available on the ability to mount memory responses upon exposure to foreign objects (*e.g.*, plastics particles) (Détrée and Gallardo-Escárate, 2018; Auguste et al., 2020).

This being a recent field of investigations, several aspects of the experimental procedure need a more thorough definition. Regarding the routes of exposure, particles are usually spiked directly in water, while pathogens (*e.g.*, viruses or bacteria) are preferentially injected into the animal. Major differences also concern the choice of the time frame between priming and challenge, often depending on the model system (from few days to months), and the protocol for immune stimulation (*e.g.*, same dose and type of agent *vs*. higher dose or different agent for challenge; priming with molecules that act like a “vaccine” and mimic the presence of a true pathogen). These issues still represent the major hurdles that affect comparison among different studies.

Concerning the techniques used to quantify the memory responses, new methods such as -omics approaches, with transcriptomics being the most common, can offer a large coverage of the putative mechanisms involved in innate immune memory. However, the use of more classical/conventional methods (*e.g.*, haemolymph functional parameters, quantification of selected immune-related genes) has proven useful to unravel the biological processes regulated upon challenge.

### Tunicates

#### Immunity and Immune Response Components

Tunicate immune defence relies on blood cells endowed with phagocytic and/or cytotoxic activity and on humoral factors mediating chemotaxis, opsonization and inflammation (Pinto et al., 2003; Franchi and Ballarin, 2014). The sites of immune recognition and response in ascidians are the pharynx, the haemolymph, and the digestive tract. The pharynx is involved in respiration and food collection, and, with its abundance of mature and immature blood cell types, is also considered the main haemopoietic site (Ermak, 1976). The ascidian haemolymph, flowing through a lymph-like circulatory system, encompasses nutrients, haemocytes, and molecules involved in the immune process. Haemocytes, engaged in immune responses (immunocytes) (Cima et al., 2016), include phagocytes and cytotoxic cells, express most of the pattern-recognition receptors and actively transcribe genes required for immune defence (Shida et al., 2003).

#### Routes of Exposure

For immunotoxicity assessment, animals should be exposed to the agents under study (chemical contaminants, particles, microplastics) in controlled conditions. Since tunicates are filter feeders, the conditions of exposure are crucial for obtaining meaningful results, and should encompass a relevant concentration of the contaminant in the water at any given time and a relevant exposure time, in order to reproduce the conditions occurring in real life (Galloway and Depledge, 2001).

#### Systemic Immune Responses

The ascidian digestive tract displays a constitutive level of immune-related transcripts that are upregulated in response to bacterial lipopolysaccahride (LPS) (Parrinello et al., 2016), suggesting the direct involvement of the gut in the recognition and clearance of foreign material. This assumption is confirmed by the presence of transcripts of genes for Toll-like receptors (TLRs), MBLs and MBL-associated serine proteases (MASPs) in both the stomach and the intestine, in addition to haemocytes circulating in the alimentary tract (Sasaki et al., 2009; Skjoedt et al., 2010). Variable region-containing chitin-binding proteins (VCBPs) are also secreted in the gut lumen, hypothetically for stabilising the commensal gut microbial flora (Dishaw et al., 2011).

#### Methods and Assays for Assessing Cellular Responses

The approaches to analyse the immune defence activities of animals exposed to various agents *in vivo* include assessments on their haemocytes and haemolymph, starting from the hematopoietic proliferation index, *ex vivo* functional assays and transcriptomics (on haemocytes), biochemical and enzymatic tests (on cell-free haemolymph). Studies of innate/inflammatory response induced by LPS have shown an upregulation of cytokine-like genes such as TNFα-like (Parrinello et al., 2008) and IL-17 (Vizzini et al., 2015) in both the pharynx epithelium and haemocytes. Depending on the species, immunocytes can produce several defensive molecules, such as complement components (Franchi and Ballarin, 2014), hydrolytic enzymes (Cima et al., 2016), the precursor form of phenoloxidase (proPO) (Franchi and Ballarin, 2016), histamine and heparin (García-García et al., 2014).

Ascidian phagocytic and cytotoxic cells have been exploited to test *in vitro* or *ex vivo* the effect of biotic and abiotic stimuli (Menin et al., 2008; Franchi et al., 2014; Cima and Varello, 2020; Valsesia et al., 2021). While the use of cells under *in vitro* conditions is a valuable alternative to test potential toxicants, at present no long-term cell culture systems have been established for most marine invertebrates (Rinkevich, 2005; Cai and Zhang, 2014). A first attempt of long-term cell culture was performed with *Botryllus schlosseri* blood cells, which can be cultured for several months but that lost reactivity over time (Rinkevich and Rabinowitz, 2000). Successful long-term cell culture was obtained from pharynx explants of the solitary ascidian *Styela clava*, which could maintain viability and proliferative capacity for up to 80 days (Raftos et al., 1990; Sawada et al., 1994), whereas circulating haemocytes remained viable in culture for a shorter period (only 18% viability after 20 days, no proliferation) (Ermak, 1982), although they could still release haemagglutinin (Arizza et al., 1997).

#### Immune Memory

Among tunicates, the ascidian *C. robusta* is a good model for assessing the establishment of immune memory. A classical *in vivo* experimental design consists of the sequential exposure of *C. robusta* adult individuals to a priming stimulus followed by a period of resting to return to basal conditions, and a challenge with a second stimulus in homologous or cross-stimulation (Melillo et al., 2019). Exposure of *C. robusta* to microbial agents induces a reaction that primes animals for developing a different (expectedly more protective) response to subsequent challenges, showing the effective establishment of an immune memory. The endpoints of immune activities measured are the same as mentioned above, *i.e.*, the frequency of haemocyte subpopulations, the *ex vivo* functional assays on haemocytes (phagocytosis) and the regulation of transcription of immune-like genes in the pharynx and gut. As different stimuli have been used in homologous and cross-combination, it was possible to identify different mechanisms establishing immune memory and whether they were priming-specific, challenge-specific, or non-specific.

Other assays can be applied for assessing the establishment of immune memory, such as degranulation of granular haemocytes, and extent of C3 activation, which is a measure of inflammatory anaphylatoxin (C3a) and oposonin (C3b) in the haemolymph (Marino et al., 2002; Giacomelli et al., 2012).

**FIGURE 2:**
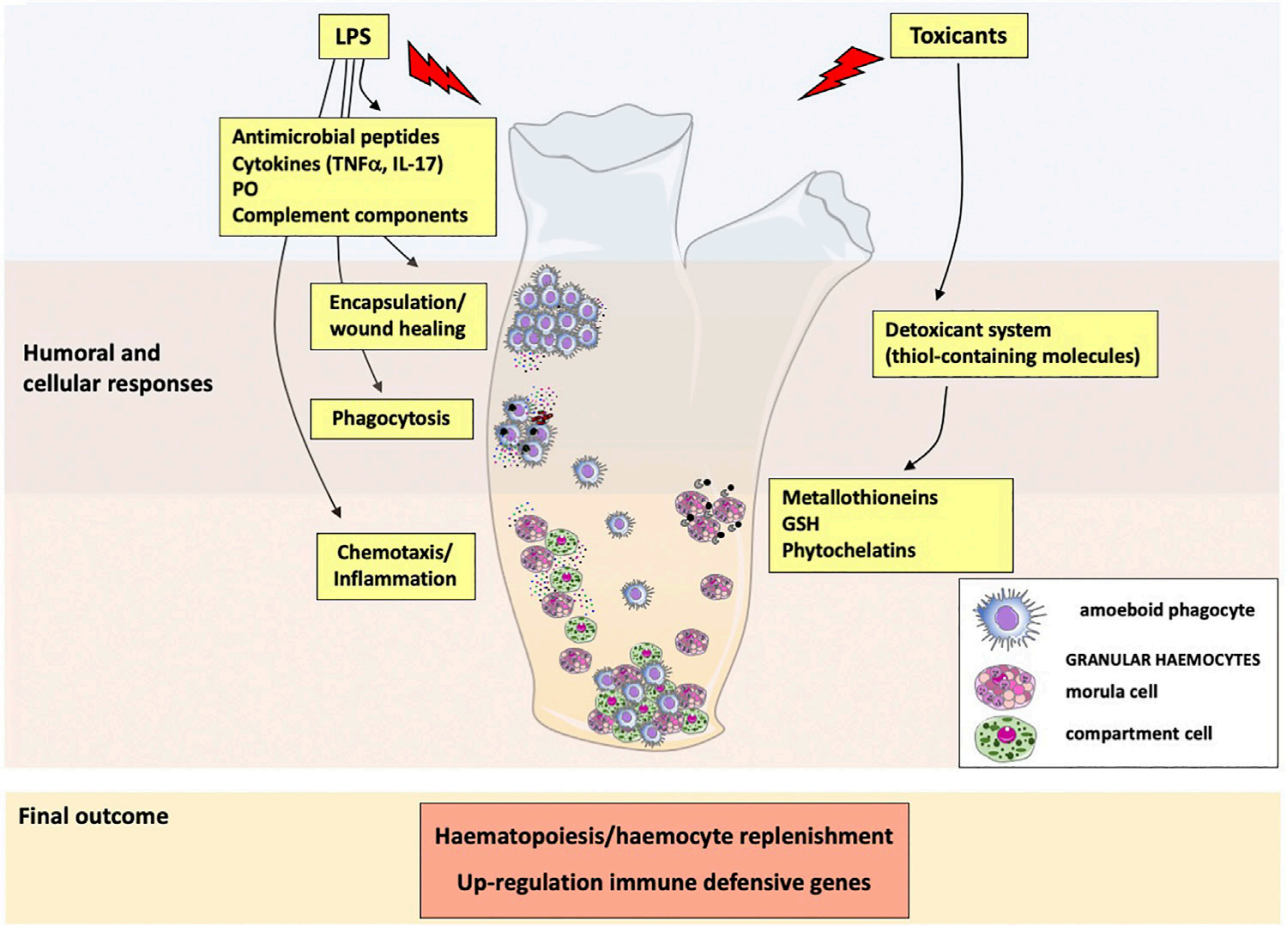
Immune responses and defensive reactions in *Ciona robusta*. Following exposure to inflammatory molecules (LPS), immunocompetent haemocytes are activated, and granular haemocytes release antimicrobial peptides, the cytokines TNFα and IL-17, phenoloxidase and complement components. These molecules participate to cellular defence activities, such as phagocytosis, encapsulation and wound healing, and recruit other immune cells to the sites of inflammation. Exposure to toxicants activates a detoxifying system (thiol-containing molecules) through the direct release of metallothioneins, GSH and phytochelatins by granular haemocytes. Exposure to inflammatory/toxic agents modulates the expression of immune-related genes and activates haematopoiesis.

Figure 2 summarises the patterns of immune response of *C. robusta* to external stimuli and toxicants.

### Sea Urchin

#### Immunity and Immune Response Components

In the sea urchin, the immune response is brought about by a heterogeneous population of freely circulating cells within all coelomic spaces, including the water-vascular system, tissues, and organs, of which macrophage-like phagocytes are the most abundant cell type (Smith et al., 2018). The sea urchin circulating cells represent a rich source of information on the physiological state of an individual. Cells feel environmental stimuli through an abundant repertoire of immune functional molecules (identified based on homology at the RNA level), including more than 700 innate pathogen recognition and 400 environmental sensing and response molecules (Goldstone et al., 2006; Hibino et al., 2006). Sea urchin immune cells can be employed as a tool for analysis of toxicity and safety prediction, and can also be used to provide useful data on the pollution status of marine ecosystems. The abundance of each cell type can change depending on health *vs*. Disease conditions and in response to an insult.

#### Routes of Exposure

The use of sea urchin immune cells for the risk assessment of long-term exposure to sources of pollution has been extensively developed and validated (reviewed in Pinsino and Matranga, 2015). Links between the sea urchin immune state and environmental factors in the field have been established at first by analysing the phenotypic profiles of sea urchin cellular subpopulations.

In the natural environment, the sea urchin immune system is continuously stimulated by diverse challenges. Thus, wild animals are immunologically more activated than those maintained in laboratory tanks. Housed animals become immunologically quiescent after months of housing (Alijagic et al., 2021a), and can be stimulated again under controlled conditions (Nair et al., 2005). To ensure the representativeness of the results using wild animals (field studies), a significant number of specimens should be examined, whereas this number could be reduced under controlled conditions. *In vivo* laboratory studies include ingestion and injection as preferential routes of exposure (Falugi et al., 2012; Pinsino et al., 2015).

#### Methods and Assays for Assessing Cellular Responses

The heat shock protein 70 (HSC70/HSP70) has been used as biomarker to assess the immunological reactions to environmental threats, including changes in temperature, pH and osmotic pressure, exposure to contaminants, UV-radiation and natural insults (Matranga et al., 2005; Pinsino et al., 2008). However, we have to consider that the HSC70/HSP70 protein level regulation is complex, and the relation between stress and HSC70/HSP70 upregulation is not linear. Thus, the use of HSC70/HSP70 as an index of stress requires a higher degree of validation. Recent developments in immunotoxicity assessment in the sea urchin imply the use of a selected panel of biomarkers encompassing both gene expression and protein levels (*e.g.*, p38 MAPK, ERK, TLR4-like, HSP70, Interleukin-6) or more global approaches using biochemical, cell biological, and -omics technologies (Pinsino et al., 2015; Migliaccio et al., 2019; Alijagic et al., 2020; Alijagic et al., 2021b). In this context, metabolism and antioxidant activity seem to represent a relevant target for immunotoxicity assessment (Migliaccio et al., 2019; Alijagic et al., 2021b).

Apoptosis assays and active caspase detection are not good prognostic tools/biomarkers for the investigation of the sea urchin immunological state *in vivo*. In general, both the number of dead cells and the level of caspase activity are low, because immune cells are capable of adjusting to environmental changes by activating robust mechanisms of protection, resistance, and immunological plasticity (Matranga et al., 2005; Rast et al., 2006). Sea urchins are known for the lack of neoplastic diseases, and immune cells can most efficiently repair hydrogen peroxide-induced damage and develop a massive antioxidant response upon stress (Reinardy and Bodnar, 2015). Live cell imaging with specific dyes, including Neutral Red (NR) and 3, 3′-dihexyloxacarbocyanine iodide, is a good tool for assessing the physiological state of subcellular organelles (*e.g.*, phagolysosome formation, endoplasmic reticulum, Golgi and vesicle membranes) (Pinsino et al., 2015). The development of optimised culture protocols resulted in a maintaining adherent phagocytes in pure stable cultures for 2 weeks, with >80% viability and full functional capacity (Pinsino and Alijagic, 2019). Based on the high similarity between sea urchin and human immune-related genes (Rast et al., 2006), sea urchin primary phagocyte cultures can be used as a human phagocyte proxy, specifically relative to innate immunity and inflammation, in line with the guidelines on the responsible use of animals in biomedical research (the 3R principle) (Herrmann et al., 2019). Sea urchin immune cells are a large source of functional molecules, which are secreted into the extracellular environment (Alijagic et al., 2019; D’Alessio et al., 2021). Methods for studying intra- and extra-cellular immunological responses encompass most of the approaches used for human primary cell cultures, including imaging, biochemistry, cellular and molecular biology, cytofluorimetry, proteomics, transcriptomics, and metabolomics (Smith et al., 2019; Alijagic et al., 2020; Alijagic et al., 2021a; Alijagic et al., 2021b; D’Alessio et al., 2021), obviously optimized for the characteristics of the system (*e.g.*, high salt concentration).

#### Immune Memory

The sea urchin anti-graft immunity is an example of a secondary immune response, primed by past experience, that increases upon a second experience (Coffaro and Hinegardner, 1997). However, how and if sea urchin immune cells possess innate immune memory is an open issue that needs attention. Therefore, no methods for assessing immune memory have been proposed yet in the sea urchin.

**FIGURE 3:**
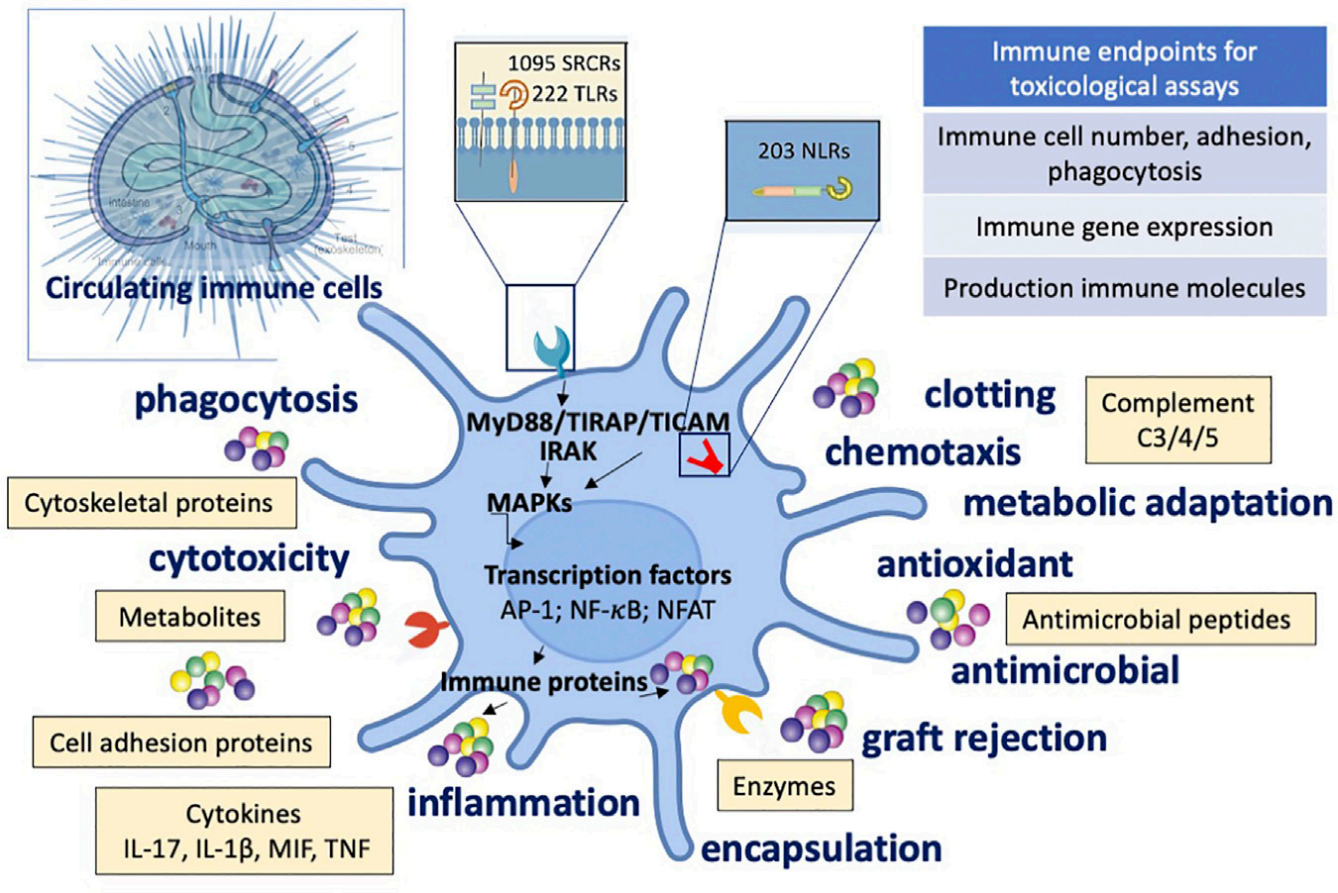
Immune defensive reactivity of sea urchin to external stimuli and toxicants. Schematic depiction of the immune functions, molecules, and signalling pathways activated during the sea urchin defensive response to threats and toxicants. Upper left: sea urchin anatomy. SRCR: Scavenger Receptor Cysteine-Rich; TLR: Toll-like receptor; NLR NOD-like receptor.

Figure 3 depicts the features of the sea urchin immune response to external stimuli and toxicants.

### Humans

#### Immunity and Immune Response Components

At variance with invertebrates, human beings possess two parallel systems of immunity, the evolutionarily conserved innate immunity and the highly sophisticated and specific adaptive immunity. Since innate immunity is the first line of defence of the organism, and the innate cells are the first that could raise an inflammatory reaction upon interacting with harmful stimuli, the majority of immunotoxicological assays are based on assessment on innate immunity/inflammation. Innate immune cells include different subsets of granulocytes (neutrophils, eosinophils, and basophils), dendritic cells, monocytes, macrophages, mast cells and innate lymphoid cells. Soluble innate immune effectors include the complement system, innate cytokines and chemokines, toxic ROS and NOS, defensins and antimicrobial peptides, among others (Kenneth and Casey, 2017). All of them participate to the development of a defensive inflammatory reaction, with monocytes and macrophages playing a major role. Recently, it has also been described that the innate immune system is able to develop an immunological memory, *i.e.*, the capacity to mount a potentiated (trained immunity) or decreased (tolerance) inflammatory reaction upon secondary challenges based on previous stimulations (Ifrim et al., 2014).

#### Methods and Assays for Assessing Immune Responses

Since the *in vivo* immunotoxicological methods are not easy to perform in a controlled fashion, analysis of innate immune system activation in humans is mainly based on *in vitro* tests. *In vitro* models have been predominantly based on transformed cell lines or primary blood leukocytes, whose reactivity to toxicants (besides survival) is mainly assessed in terms of production of inflammatory and anti-inflammatory factors, cytokines and chemokines. It is however important that the *in vitro* models can realistically reproduce the specific immunological processes that occur *in vivo* (such as innate/inflammatory reaction and the innate immune memory re-programming process), in order to obtain predictive results.

The most reliable *in vitro* models are based on human primary monocytes or monocyte-derived macrophages (Li et al., 2016). Monocytes are key inflammatory cells that come from blood into tissues during an inflammatory reaction. The use of primary human cells allows us to study the mechanisms of innate/inflammatory or memory reactions in conditions that readily translate to human responsiveness *in vivo*, as opposed to mouse models or *in vitro* models based on transformed cell lines (such as THP-1 or U937, *i.e.*, tumour cells that share some characteristics with primary monocytes; Gennari et al., 2004; Gennari et al., 2005). The use of immortalized cell lines offers several advantages: they are cost-effective, easy to use, provide a pure population of cells with unlimited proliferation, bypass ethical concerns associated with the use of human tissues, and finally provide reproducible data. However, they are scarcely relevant from a biological/functional point of view, since they may have lost many characteristics of the original cells (*e.g.*, the capacity to produce some inflammatory factors in response to challenges) and developed other activities that are absent in primary cells (*e.g.*, uncontrolled proliferation). Primary immune cells may better reproduce the behaviour of normal human cells *in vivo*, and also provide the opportunity to assess donors’ variability in immune reactivity, which is the basis for developing effective approaches of personalized medicine (Brodin and Davis, 2017).

Human innate/inflammatory reactivity can be measured with two well-established *in vitro* assays: the Whole Blood Assay (WBA) and Monocyte Activation Test (MAT) (Hartung and Wendel, 1995; Langezaal et al., 2002; Hoffmann et al., 2005; Schindler et al., 2009; European Pharmacopoeia, 2019; Hartung, 2021). The MAT in particular is a test adopted for the detection of pyrogens in pharmaceutical products and is, therefore, suitable for detecting immunotoxicity in terms of induction of inflammation. These are the simplest *in vitro* models used for assessing the direct capacity of different stimuli to activate innate responses, using as endpoint the production of the inflammatory factors (in general inflammatory cytokines) upon acute exposure (*e.g.*, 4 and 24 h). The WBA is based on the use of anticoagulated whole blood and allows us to study the global reactivity of blood cells and to assess the production of a number of different mediators, pertaining to monocytes, granulocytes, lymphocytes, platelets, *etc*. The MAT is based on the production of inflammatory cytokines (IL-6, TNFα, IL-1β) by circulating monocytes or mononuclear cells (PBMC) isolated from blood. Both assays are simple but allow for a reliable evaluation of human cell innate/inflammatory activation as well as direct toxic effects by different compounds. A further simplification can be made in the MAT test, with the use of monocyte-like cell lines instead of primary blood cells. This has the advantage of reducing variability, which may be a problem when using cells coming from different donors. However, since cell lines are not normal cells, their reactivity to stimuli/toxicants can be significantly different from that of primary cells, thereby hampering the value of the test (Okemoto et al., 2006; Lankveld et al., 2010; Andreu et al., 2017; Studholme et al., 2019). Other assays have been described for assessing changes in innate cell reactivity, based on NK cells (testing their cytotoxic capacity) and dendritic cells (DC; testing their capacity to mature) (Hymery et al., 2006; Li, 2018).

While these can be useful tools in human immunotoxicology, it should be underlined that an inflammatory reaction is an expected defensive reaction to any challenge, and it only suggests that the innate immune system is efficient. Conversely, an immunotoxic reaction can be only identified by examining other parameters, such as the rapidity of response initiation, the length of its duration, the extent of its magnitude, the balance between inflammatory and anti-inflammatory factors. Thus, the simple WBA and MAT tests are good for identifying the activation of an innate response but they fall short of predicting immunotoxic effects.

In this perspective, a kinetic evaluation of the regulation of monocyte activation and macrophage functional differentiation during an inflammatory reaction is required. *In vitro* models have been developed, using human primary cells kept in culture in conditions that reproduce the kinetic changes in the tissue microenvironmental conditions experienced by monocytes participating to inflammation (Italiani et al., 2014; Italiani et al., 2020). These models describe in a simplified but reliable fashion the kinetic development of human monocytes reactivity and its modulation during the entire course of resolving or chronic inflammation, providing the basis for understanding whether changes in their transcriptomic and proteomic profiles upon exposure to toxicants may be predictive of health hazard (*e.g.*, a shift from physiological to pathological profile) (Li et al., 2016).

Even though this approach is more accurate in reproducing the course of a human *in vivo* inflammatory reaction, thereby allowing for the predictive identification of immunotoxicity effects, it presents several limitations that make its use hardly feasible. Besides the inherent variability due to the use of primary cells from different donors (an issue common to all *in vitro* assays based on primary blood cells), the assay needs time (several days) and specialized skills. Another limitation is conceptual, since the model exclusively reproduces the reactivity of the first monocyte wave entering an inflamed tissue, while it is known that monocytes enter an inflammatory site continuously during the reaction. Also, the lack of other cells, including resident tissue macrophages (and their mechanical and biological interaction), extracellular matrix components and 3D architecture may influence the monocyte reactivity in unforeseeable ways (Jain and Vogel, 2018; Jain et al., 2019).

As a step forward in the validity of *in vitro* immunotoxicological assays, several co-culture assays are being developed and used (Leonard et al., 2010; Noonan et al., 2019; Drasler et al., 2020; Karri et al., 2021). For example, an *in vitro* model of the inflamed intestinal mucosa, encompassing a three-dimensional co-culture of human intestinal epithelial cells (the cell line Caco-2) with human primary monocytes and monocyte-derived dendritic cells, can be used for assessing the effect of drugs and toxicants on gut inflammation (Leonard et al., 2010). While many of these models aim at a more thorough evaluation of immune reactions within the context of a tissue, still they cannot reproduce the complexity of cell-cell and cell-stroma chemical and mechanical interactions that take place *in vivo* in healthy or disease conditions. An advancement in this direction is the development of “organs-on chip”, *i.e.*, 3D tissue cultures set in microfluidic systems (Ulleras et al., 2005). Organs-on-chip use microfabrication technologies to replicate the tissue microenvironment in its 3D microarchitecture (*i.e.*, the spatial distribution of different cell types), its biochemical microenvironment (including chemokine, growth factor and nutrient gradients) and its mechanical microenvironment (*e.g.*, mechanical compression, cyclic strain and shear stress) (Bhatia and Ingber, 2014). Organs-on-chip can reproduce the microenvironment of different organs (*e.g.*, gut-on-chip; Irimia and Wang, 2018) and in different conditions (*e.g.*, inflammation-on chip; Kim et al., 2016), thereby allowing for realistically monitoring human immune cell reactivities (Herrmann et al., 2019). In general, however, when building co-culture models that encompass immune cells, caution should be taken as such cells may react against the other cells in the co-culture (in particular if they are transformed or tumour cells), thereby creating a background sactivation state that would affect the immunotoxicological assessment.

**FIGURE 4:**
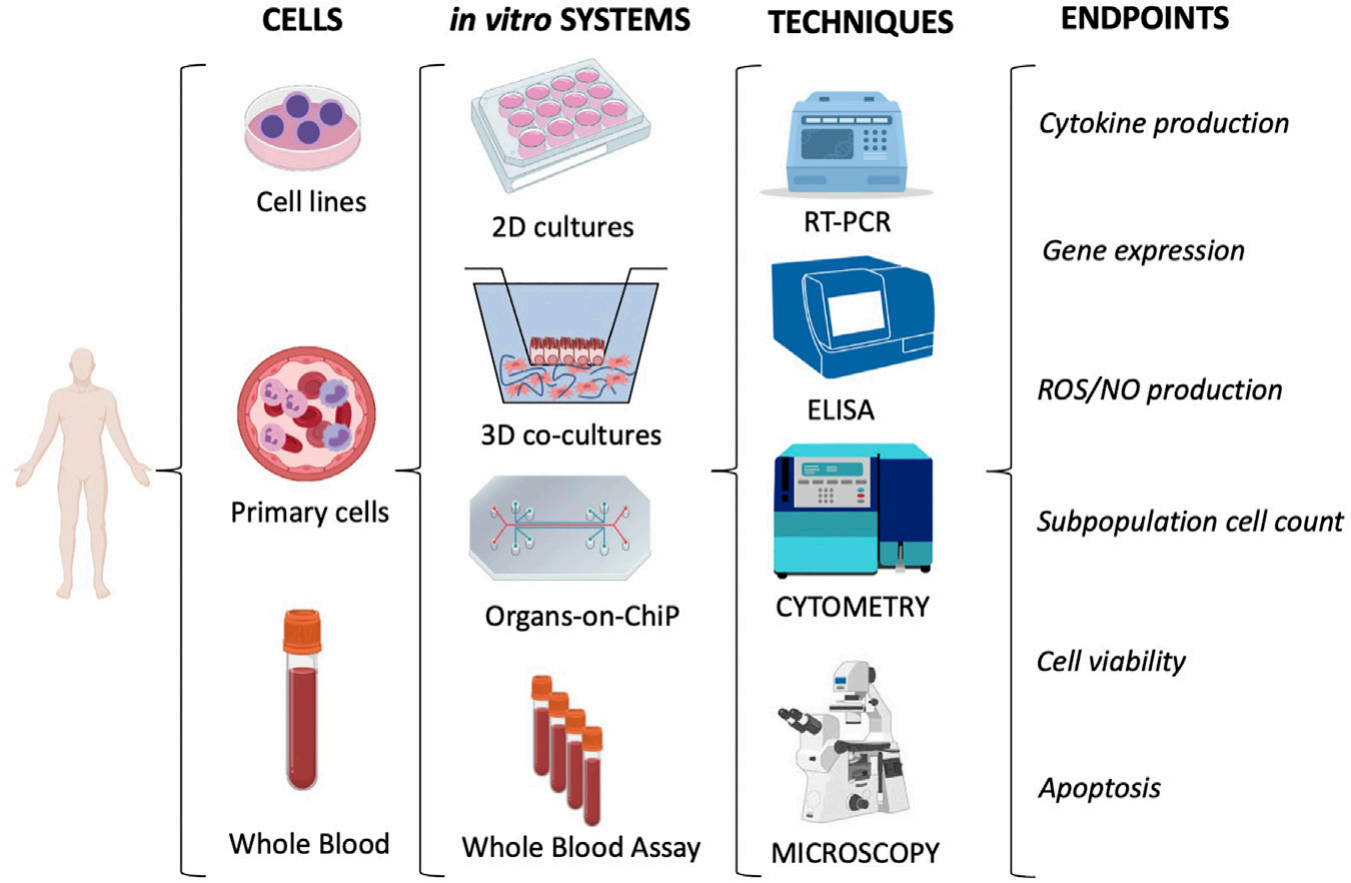
Immunotoxicity evaluation in human cells. The most used *in vitro* models/systems are based on cell lines or primary cells (*e.g.*, leukocytes isolated from whole blood). They encompass conventional 2D cultures, 3D co-cultures and the more innovative Organs-on-ChiP arrays. The whole blood can be considered as a “ready-to-use” system. 2D cultures with monocytic cell lines (*e.g.*, THP-1 or U937) or with the more reliable primary cells (e.g., monocytes, NK or DC) are mainly used to measure the inflammatory reactivity of innate immune cells. Conversely, the Whole Blood Assay (WBA; based on the use of anticoagulated whole blood) allows us to study the global reactivity of blood cells. Overall, the immunotoxicity is evaluated by measuring several biological and functional endpoints through different evaluation techniques. The main techniques and endpoints are shown.

A summary of the immunotoxicity methods that can be adopted for assessing effects on human cells is reported in Figure 4.

#### Immune Memory

The mechanisms underlying the innate immune memory observed *in vivo* (tolerance or potentiation) can be studied *in vitro* using cell-based models based on human peripheral monocytes, primed in culture with a given stimulus (*e.g.*, microbial products) for a short period of time (usually 24 h). Subsequently, cells are incubated for 7 days in culture medium without any stimulation, a resting period in which the functional programme of the cells returns to their steady-state basal level. The return to steady-state functionally quiescent state is particularly relevant when examining innate memory, to prevent mistakenly evaluating adaptation or differentiation mechanisms (Divangahi et al., 2021). If the primary stimulus induces transcriptional and epigenetic changes in cells, after the resting period and the extinction of the primary response they will show a different reaction to homologous or heterologous secondary stimuli (Ifrim et al., 2014; Madej et al., 2017; Swartzwelter et al., 2020), which can be assessed as an increased or decreased production of inflammatory and anti-inflammatory factors.

The tolerance and potentiation memory responses assessed *in vitro*, however, cannot be taken as predictive of increased or decreased resistance to the infections or diseases *in vivo*, as they only reflect a decrease or increase in the reactivity of monocytes to stimuli, *i.e.*, a re-programming of their responsiveness (Italiani and Boraschi, 2017). Since the functional outcome of innate reactivities is a balance between immunostimulatory/inflammatory responses and feedback mechanisms that control/down-regulate inflammation, a more predictive assessment of effects on innate memory implies examining the simultaneous production of inflammatory and anti-inflammatory factors, which would allow for predicting whether memory will induce a more inflammatory or less inflammatory response profile relative to the primary exposure to stimuli/toxicants (Della Camera et al., 2021).

Notably, with this approach it is possible to define a personalised memory profiling, which can allow defining the individual effects of toxicants/contaminants on innate memory and predict the adequacy of protective responses to future challenges (*e.g.*, infections). During the development of a new immunization strategy, *ex vivo* stimulation assays could be used for studying innate immune priming and help assessing the effectiveness of a vaccine from the point of view of capacity for non-specific innate/inflammatory response amplification.

The assays described above are mainly based on monocytes, but they are adaptable to other types of innate cells such as macrophages, mast cells and Innate Lymphoid Cells (ILCs), although no specific immunotoxicity methods have been proposed yet to assess innate memory with these cells.

## Major *Ex Vivo/In Vitro* Immunotoxicity Assays

Hereafter we report a list and brief description of the main assays that are applied, in the different animal species, for measuring immunotoxic effects. Table 4 summarises the immunotoxicity endpoints and the assays for addressing them across species, underlying commonalities and differences. For invertebrates, most of the assays can be applied for *in vitro* testing or *ex vivo* evaluation after *in vivo* exposure, with only limited adaptation of the existing protocols.

**TABLE 4:** SWOT analysis of the cross-species assays/endpoints for immunotoxicity assessment.

Endpoint	Assay	Strengths	Weaknesses		Opportunities	Threats
Cytotoxicity assays						
Cell viability	Trypan blue	• Fast, cheap	• Dye not stable over time		• Application to a large panel of species	• The dye is toxic
		• Reproducible	• Require fresh haemocytes			
		• Few materials required	• Viability may be overestimated (apoptotic cells appear viable)			
	MTT	• Robust	• Used on adherent cells		• Protocols adaptable to a large panel of species	• No clear relationship between metabolic activity and cell number
		• Easy to use	• Cell detachment during supernatant removal			
		• High sensitivity	• Chemical interference			
		• Gold standard for cytotoxicity testing	• It only measures metabolically active cells			
			• Kits are expensive			
	Lysosomal Membrane Stability (LMS)	• Vital staining	• Applies only to granulocytes		• Use in standardised protocols and guidelines	• Limited to adherent cells
		• Single cell information			• Additional information on cell shape	• Requires training for appropriate identification of parameters
	LDH release	• Robust	• Serum has LDH activity		• Provides information on cell membrane integrity and irreversible cell death	• It does not include other cytotoxic features (*e.g.*, cytostasis, apoptosis)
		• Reliable	• Kits are expensive		• Mostly used in human cells but applicable to invertebrates	
		• Simple evaluation				
Apoptosis	Detection of apoptosis-related changes (*e.g.*, Annexin V, TMRE)	• Provides early signs of apoptosis	• Low solubility in seawater for some probes		• Common probes for different species	• Can be toxic for cells
		• Single cell information	• Some interactions of the probes with other cell membrane components can create artefacts		• Can add mechanistic information on the toxicity data	• High cost
			• Photo-bleaching			• Sensitive to changes in the exposure medium (*e.g.*, salinity, pH)
Genotoxicity DNA damage	Comet assay	• Sensitivity for detecting low levels of damage	• Only applicable to fresh cells		• Applicable to many species	• Genotoxic damage is not to be taken as health risk, as it refers to some cells rather than to the entire organism (unless when affecting some specific cell types)
		• Requires small number of cells per sample	• Long sample preparation process			
			• Does not distinguish between different types of damage (single/double strand breaks, apoptotic fragments, *etc*.)			
			• Does not identify mutations			
Evaluation of cell number and phagocytic capacity						
Total and subpopulation cell counts	Microscopy, Flow Cytometry	• Provides a general picture of the whole population	• Requires specialised expertise		• Allows to compare samples between different conditions (activation phases, health status, season, etc.)	• Method standardization can vary among labs
		• Provides number and frequency of subpopulations	• Requires special instrumentation (cytofluorimeter)			• Changes may be due to or masked by external factors
		• Accurate single cell phenotypic characterization with analysis of multiple markers				• Affected by seasonality (in marine invertebrates)
Phagocytosis	Fluorescent bacteria or zymosan particles	• Simple recognition and cell counting (fluorescent microscopy)	• Apply mainly to granulocytes/adherent cells (microscopy)		• Applicable to many species	• Bell-shaped dose response curve
		• Fast (cytofluorimetry)	• Time consuming (microscopy)		• Allows to identify cell subpopulations	• No info on phagocytosis rate unless the entire cell population is sampled
		• Small sample volume required	• Requires special instruments (cytofluorimeter, fluorescence microscope) and skilled personnel			
Production of defensive and immune-related molecules						
Lysozyme release	Hydrolysis of *M. lysodeikticus*	• Can be evaluated in serum		• Kinetic measurements that require temperature control	• Applicable to many species	• Affected by seasonality in invertebrates
		• Low cost				
Reactive oxygen species (ROS)	Cytochrome C reduction	• Efficient in evaluating extracellular ROS release		• Cyt C can interact with other enzymes	• Applicable to many species	• Affected by seasonality (for marine organisms)
		• Fast		• High cost		• Low detection for ROS generated in organelles
	Use of fluorescent probes (*e.g.*, H_2_DCFDA)	• Intracellular detection		• Cell auto-florescence can interfere with the ROS-generated signal	• Suitable for respiratory burst	• Does not distinguish between different ROS
		• Cell permeable probes				
NO production	Griess reaction	• Provide a quantifiable reaction		• High limit of detection (µM range)	• Can be further modified or completed to detect other nitrogen species	• Susceptible to contamination
		• Low cost		• Limited to nitrite detection		• Reagent can interfere with serum components
	Use of fluorescent probes (*e.g.*, DAF-FM/DAF-FM diacetate)	• Provide details/localization in cell		• Low cell membrane permeability	• Can be used for different species	• Can react with other NO metabolites
		• Low detection limit (nM range)				
Immune-related gene and marker expression						
Immune gene expression	Transcription by qRT-PCR	•Quantitative gene expression levels		• Information limited to the examined genes, no protein/functional information	• Informative implementation of functional data	• Limited primer availability for some organisms
				• Normalization required for quantitative measurements		
Unbiased immune marker detection	Transcriptomics, proteomics, lipidomics, metabolomics	• Analysis of changes in activation pathways and protein repertoires		• Excess of data that may be difficult to handle properly	• Can offer a large coverage of the putative mechanisms involved in immunotoxicity	• Should be combined with functional assays
		• Applicable at the single cell level for assessing population functional heterogeneity		• Expensive	• Highly informative for organisms for which other methods are unavailable (*e.g.*, invertebrates)	
				• Specialised skills and instrumentation required		

### Cytotoxicity Assays

Trypan blue vital dye is a useful procedure for estimating alterations in membrane permeability associated with cell death in mammals (Louis and Siegel, 2011). The same procedure is often adopted for the evaluation of immune cell viability in different invertebrate species (Rocha et al., 2014; Pinsino et al., 2015; Senior and Titball, 2020). The main drawbacks of this technique are the toxicity of trypan blue, which limits the possibility of using it to few minutes after staining, and the high number of false negatives (*e.g.*, lack of detection of cells already engaged into the apoptotic pathway as they still have intact membranes). In addition, in marine animals the use of trypan blue is undermined by its poor solubility in Artificial Sea Water/Filtered Sea Water at any concentration. Valid alternatives for evaluating cell viability are Ethidium Bromide, Propidium Iodide and similar DNA-binding molecules (Raftos and Hutchinson, 1997; Chan et al., 2015). In dissociated mussel gill epithelial cells and haemocytes and adult sea urchin immune cells, early signs of stress, viability and toxicity can be evaluated by standard cytotoxicity assays in 96 microwell plates generally used for human cells (*e.g.*, MTT assay; Mosmann, 1983; Präbst et al., 2017) optimized for application to invertebrate cells (Katsumiti et al., 2015; Alijagic et al., 2019; Pinsino and Alijagic, 2019; Alijagic et al., 2020).

As already mentioned, these assays allow for the quantitative evaluation of dead cells and are applicable to both invertebrate and vertebrate immune cells.

For mammalian cells, another common way for assessing cell integrity is by measuring the amount of the cytoplasmic enzyme lactate dehydrogenase (LDH) released by cells upon exposure to toxicants. LDH presence in culture supernatants correlates with the number of damaged/dead cells, and it can be quantitatively measured with colorimetric assays based on LDH-dependent generation of coloured products (Fotakis and Timbrell, 2006).

Lysosomal membrane integrity is a general common target for environmental stressors in all eukaryotic organisms. In mussels, the lysosomal membrane stability (LMS) correlates with other stress biomarkers, thus representing a good diagnostic endpoint of the individual health status (Moore et al., 2004). The LMS assay is a standardized testing procedure (OSPAR Commission, 2013), based on the ability of viable cells to incorporate the supravital dye NR in the lysosomes, which provides a quantitative estimation of the number of viable cells in a culture. Immune cells stained with NR accumulate it in intact lysosomes. The NR release from leaking lysosomes or fusion processes can be monitored microscopically over time as NR retention time (Moore et al., 2004), or spectrophotometrically by extracting with an acidified ethanol solution the dye retained by viable cells at the end of the incubation time (Borenfreund and Puerner, 1985; Katsumiti et al., 2015). The percentage of viable cells in short-term cultures is calculated by counting cells retaining red lysosomes out of total cells.

This assay is very accurate, and it can be used to measure cell replication, cytostatic effects or cell death depending on the seeding density. However, it does not provide information on responses of agranular haemocytes (which do not uptake the dye) in mussels.

Apoptosis assays detect and quantify the cellular events associated with programmed cell death. The most used methods for assessing apoptosis (both in invertebrates and in human cells) include the use of: 1) nuclear dyes (as acridine orange) able to detect chromatin condensation; 2) tunel assays or other DNA fragmentation detection tests, for detecting DNA fragments associated with extensive damage to chromatin, and 3) annexin V detection, *e.g.*, using a luminescent/fluorescent assay that monitors changes in the distribution of phosphatidylserine in the plasma membrane during apoptosis (Koopman et al., 1994; Collins et al., 1997; Muganda, 2016; Auguste et al., 2018; Cima and Varello, 2020; Sendra et al., 2020). In addition, in *Mytilus* haemocytes and sea urchin immune cells apoptosis can be evaluated with the comet assay (DNA damage) (Canesi et al., 2014; Rocha et al., 2014), DNA staining with Hoechst 33258 (Matranga et al., 2006), detection of caspase3/7 (Alijagic et a., 2019), mitochondrial membrane potential (tetramethylrhodamine ethyl ester perchlorate, TMRE) (Auguste et al., 2018; Sendra et al., 2020).

In general, all these assays can be used for human and invertebrate cells with little modifications.

### Cell Spreading Assay

The spreading index is a useful measure of cells directly involved in the immune defense in invertebrates (Pinsino and Alijagic, 2019; Cima and Varello, 2020). Briefly, after adhesion to a substrate, cells are incubated with various concentrations of a toxicant, then either fixed or not (depending on the species) and counted. The spreading index is expressed as a ratio between spread/amoeboid adherent cells and total adherent cells.

This assay is used for tunicates and sea urchins, but is not adopted for human cells.

### Haemopoietic Cell Index

In tunicates, cell proliferation has been adopted as an index of haemopoiesis by measuring the incorporation of labelled nucleosides (BrdU or EdU) in cultures of small organ pieces containing haemopoietic tissues, isolated haemocytes (either treated *in vitro* or coming from treated animals), or whole organisms (Raftos and Cooper, 1991; Raftos and Hutchinson, 1997). Fluorescent labelling can be detected cytofluorimetrically in parallel to size/granulosity-based evaluation of lymphoblast frequency as a marker of haemopoiesis (Raftos and Hutchinson, 1997).

In bivalves, current knowledge on immune stem cells, their location and their differentiation pathways is still limited, therefore these parameters are not usually considered for immunotoxicty testing*.* Only some proliferation and differentiation markers can be assessed in circulating haemocytes (Cima and Matozzo, 2018).

Likewise, circulating immune cell proliferation in adult sea urchins is very limited (detectable in less than 10% of the circulating immune cells), as it is low the expression level of genes involved in haemopoiesis (Golconda et al., 2019). Thus, the haemopoietic cell index is not a good prognostic tool of sea urchin immunotoxicity.

The haemopoietic cell index on circulating immune cells is not applicable to human circulating immune cells.

### Degranulation

Degranulation is a defensive process in which cytoplasmic granules release their content (*e.g.*, amines, antimicrobial peptides, hydrolytic enzymes and other cytotoxic or bioactive mediators). In tunicates, degranulation can be detected by measuring histamine release by ELISA in short-term assays after *in vitro* stimulation of haemocytes, collected from animals exposed *in vivo* to toxicants. Alternatively, the fraction of haemocytes containing histamine could be detected by flow cytometry, after intracellular histamine staining (García-García et al., 2014).

Lysozyme release in the extracellular medium (or in the soluble serum fraction in *in vivo* experiments) can be measured using a simple spectrophotometric assay, which follows the hydrolysis of *Micrococcus lysodeikticus* over time. When running this assay in mussels, seasonal fluctuations in basal lysozyme activity must be taken into account (Ciacci et al., 2009b). Lysozyme release occurs in human cells as well, and the enzyme can be also detected in human biological fluids (Ragland and Criss, 2017).

In sea urchins, the naphthoquinone pigment echinochrome A is released from the cytoplasmic vesicles of red amoebocytes in the coelomic fluid upon infection, and its release is measured by UV/Vis spectrophotometry (Coates et al., 2018).

In humans, neutrophil degranulation is a marker of an ongoing inflammatory reaction and it can be detected by measuring the release of preformed mediators (as described above) and also by measuring the cell surface expression of CD63, CD66b and CD35 (specific markers of azurophilic, specific/gelatinase and secretory granule) by flow cytometry (Babin et al., 2013). The release of neutrophil extracellular traps (NETs) upon toxicant-induced neutrophil activation or death can be evaluated by a live cell imaging method (Keshavan et al., 2019). Degranulation of basophils and eosinophils is also used for assessing the toxicity of various agents, and is generally measured using cell lines or blood cells from patients with eosinophilia or basophilia and assessing the stimulus-induced *in vitro* release of pre-formed mediators (*e.g.*, histamine, Major Basic Protein) (Masilamani et al., 2017; Fettrelet et al., 2021).

Thus, these assays are used with little modifications across most animal species.

### Phagocytosis

The phagocytic ability of invertebrate immune cells can be evaluated in terms of type and quantity of particles internalized, as well in the rate of the phagocytic process, which is mediated by complex molecular processes. A variety of stimuli (contaminants and microorganisms) can either enhance phagocytosis and increase its efficiency or block/inhibit the phagocytic machinery, depending on the type of stimulus and experimental conditions. Two different *ex vivo* phagocytosis assays are used to measure the immune cell capacity to uptake and kill bacteria: 1) phagocytosis of bacteria (Melillo et al., 2019) and 2) phagocytosis of beads (Choi et al., 2010; Pinsino and Alijagic, 2019). The analysis of phagocytosing cells can be done at the single cell level by light microscopy (Pinsino et al., 2015; Melillo et al., 2019; Pinsino and Alijagic, 2019) or at the cell population level by flow cytometry (García-García et al., 2014), depending on the dye used for labelling bacteria/beads and on the use of cells in suspension or adhered to glass/plastic surfaces. With cells adherent to a slide, phagocytosis is quantified by counting the number of cells engulfing bacteria or beads under a confocal microscope. In *Mytilus*, the easiest method for evaluating phagocytosis is the microscopic observation of uptake of NR-labelled zymosan particles by adherent haemocytes (Pipe et al., 1995). In response to contaminants, this parameter usually shows a bell-shaped dose-response curve, with stimulation at low contaminant concentrations followed by inhibition at higher concentrations. However, since different immune cell subpopulations show different phagocytic and adhesion capacity, flow cytometric evaluation on cells in suspension can be used for the simultaneous identification of cell subpopulations and the quantitative evaluation of their capacity to uptake fluorescent particles/bacteria (Sendra et al., 2020).

In humans, the phagocytic assay is used to evaluate the functional ability of macrophages, neutrophils or monocytes, to uptake extracellular materials, using bacteria or other particles conjugated with fluorescent dyes (*e.g.*, FITC-labelled latex beads). Phagocytic activity can be assessed either by counting phagocytosing cells using a fluorescence microscope or measuring fluorescence intensity by flow cytometry (Sharma et al., 2014; Lehnert et al., 2021).

Two types of measures can be obtained, the phagocytic rate (number of phagocytosing cells within the total phagocyte population), and the phagocytic index (number of bacteria/beads phagocytosed per phagocytosing cell).

The phagocytosis assays can be used, with little modifications, for cells of most animal species.

### Production of Defensive Biomolecules and Enzymes

Production of reactive oxygen species (ROS) and anti-oxidant molecules by immune cells is among the most important parameters for assessing immune defensive activation (Canesi et al., 2017; Ghezzi et al., 2017; Barbosa et al., 2018; Migliaccio et al., 2019). In mussels, the level of ROS can be evaluated both in the intracellular compartment and also release in the extracellular medium (*i.e.*, the haemolymph). Harmful hydrogen peroxide cellular levels are minimized by the enzyme glutathione peroxidase using glutathione (GSH) as a reductant. Assays for measuring GSH content are commonly used in toxicological studies, including in invertebrates, both *ex vivo* and *in vitro*. In *ex vivo* experiments, whole animals are treated with potential toxicants, then immune cells are collected and stained with chlorobimane, a fluorescent dye specific for GSH. The observation can be done under a fluorescent microscope. In *in vitro* experiments, adherent cells are stained soon after treatment with toxicants. The quantification of intra- and extra-cellular ROS production can be easily evaluated using simple spectrophotometric techniques (*e.g.*, cytochrome C or NTT reduction) (Pipe et al., 1995).

Among superoxide indicators, dihydroethdium and 2',7'-dichlorodihydrofluorescein diacetate (H_2_DCFDA) are commonly used to evaluate ROS formation in cells in culture, as well as in immunocompetent tissue extracts. Both compounds, in contact with ROS, generate a highly fluorescent product detectable by a photodetector or by fluorescence microscopy (Katsumiti et al., 2015). It should be noted that some confounding factors can influence the measurement, like the interference with some contaminants and cytochrome c binding, or the auto-fluorescence observed, for instance, in *Mytilus* haemocytes. The use of flow cytometry can help in overcoming some of these problems.

In addition to the above methods, the oxidative burst in human phagocytes can also be detected by oxidation of dihydrorhodamine (DHR) to a fluorescent product detectable by flow cytometry (Chen and Junger, 2012). The nitroblue tetrazolium (NBT) dye test is another useful test to measure the human phagocyte oxidative functions, which is based on the oxidation of the colourless NBT to a deep blue product, which can be quantitatively detected spectrophotometrically. Microscopical examination can allow for the identification of the fraction of NBT-positive cells, which contain blue deposits (Bellinati-Pires et al., 1992).

Nitric oxide (NO) is a highly cytotoxic and microbicidal molecule that is actively produced during inflammatory processes. In *Mytilus*, all haemocyte subpopulations can produce NO, and the presence of NO synthase was recorded in haemocytes activated by different several stimuli (García-García et al., 2008). NO production can be evaluated by quantification of nitrite (NO_2_−) by the Griess reaction (Tafalla et al., 2002; Giustarini et al., 2008) or by flow cytometry using a NO-sensitive intracellular indicator (DAF-FM/DAF-FM diacetate) (García-García et al., 2008; Laurent et al., 2015).

In invertebrates, the activity of the enzyme phenoloxidase (PO) is a typical parameter measured for the evaluation of immune response. PO is an oxidative enzyme with cytotoxic activity, involved in defence reactions. The PO production in haemocyte lysate supernatants can be measured spectrophotometrically by recording the formation of dopachrome from L-Dopa. The same activity can be assessed cytochemically after adding L-Dopa to fixed haemocytes (Smith and Söderhäll, 1991; Cima and Varello, 2020). In bivalves, PO is measured in oysters and clams (in which PO activation is evident) using the same approach described above (Gagnaire et al., 2004). In *Mytilus*, PO is not routinely measured, although they are few protocols available (Renwrantz et al., 1996).

The ROS and NO assays are used for human and invertebrate cells with little modifications, while the PO assays are specific for invertebrates.

### Expression of Immune-Related Genes and Production of Immunoactive Molecules

A notable difference in the immunotoxicity assays in invertebrates and mammals (in particular human beings) is due to the availability of reagents for protein detection and functional assessment.

For invertebrates, most of our knowledge of immunity relies on gene expression and homology with mammalian genes (Pinsino et al., 2015; Milutinovic and Kurtz, 2016; Migliaccio et al., 2019; Alijagic et al., 2020; Alijagic et al., 2021b). Transcript analysis by qRT-PCR is routinely used to quantify transcription of a set of selected immune-related genes, such as lysozyme, Toll-like receptors, antimicrobial peptides, fibrinogen-related proteins, complement components, etc. (Auguste et al., 2020). The choice of target genes often depends on the type of sample (cells or tissues) and stimulus, experimental conditions and associated function, and have the aim to correlate changes in gene expression to the few functional immune parameters that can be assessed (cell spreading, phagocytosis, degranulation, release of ROS/NOS/enzymes). Alternatively, hypothesis-free genome-wide transcriptomics and other -omics technologies can be applied. Although they are not yet routinely used for immunotoxicity testing in invertebrates, the availability of the full genome sequence of sea urchin (Sea Urchin Genome Sequencing Consortium, 2006), *Ciona intestinalis* (Dehal et al., 2002) and, recently, of *M. galloprovincialis* (Gerdol et al., 2020) makes the application of genome-wide assessments a realistic possibility in the near future. However, in order to understand the environmental relevance of any change in immune homeostasis, a combination of such technologies with functional assays is still required (Balbi et al., 2021).

For the human system, a much wider testing strategy can be applied, compared to invertebrates, because of the availability of a plethora of specific tools, in particular monoclonal antibodies and recombinant proteins, which made possible the development of highly specific and high content assays for intracellular detection and imaging, cytofluorimetry, ELISA, CyTOF and other cytochemical detection methods. Available monoclonal antibodies can assess the quantitative levels of soluble factors (such as cytokines, chemokines, growth factors, complement components, antimicrobial peptides) and cell-associated markers (*e.g.*, adhesion proteins, receptors, membrane and intracellular markers of differentiation, maturation or activation), thereby allowing for an accurate evaluation of subtle effects on immune cell homeostasis. The stable characteristics of monoclonal antibodies also ensure a better reproducibility of results. In addition, we have available many recombinant immunoactive proteins (cytokines, chemokines, growth factors, complement components, receptors, signalling molecules, *etc.*). Thus, in human cells exposed to toxicant, we can assess the expression, production and function of immune-related factors (such as cytokines and their receptors) with a plethora of assay systems and functional methods, including high-throughput high-content assays and hypothesis-free -omics technologies (Li and Xia, 2019; Karkossa et al., 2020).

## Conclusion

Immunotoxicity methods and protocols for assessing the effects of toxicants on innate inflammatory responses have the advantage that innate immunity is highly conserved across evolution. This means that it is possible to identify mechanisms that go beyond adaptation to different environments (terrestrial, marine, freshwater) and are retained with little variation from invertebrates to human beings. Thus, relative to selected common functions/immune mechanisms, we can identify human proxys among inverterbates. Studying such mechanisms *in vivo* in invertebrates can provide toxicological profiles transferrable to the human situation, with the advantage that, since invertebrates do not possess adaptive immunity, the pure effects on innate immunity/inflammation can be assessed. The recently re-discovered mechanism of innate memory however adds complexity to the immunotoxicological evaluation, because this is highly influenced by the previous “immunobiography” of individual subjects, and therefore less reproducible in invertebrates that usually are subjected to a limited number of environment-specific challenges. A personalized assessment of innate memory reactivity is therefore foreseeable, which can take advantage of the more recent technological advancements. Indeed, it is now possible to assess stimulus-induced changes on single immune cells, using approaches such as scRNA-seq, scATAC-seq, sc proteomics and sc metabolomics, which can analyse, within a cell population, the effects on each individual cell in terms of transcripts, epigenetic modifications, protein production and metabolic changes. High-throughput/high-content screening (HTS/HCS) assays and instruments can also help in assessing the immunotoxic effects of several agents in terms of changes in multiple cell parameters. Several of these HTS/HCS technologies (such as CyTOF, O-link, CITE-seq, Opera Phenix) are currently applied to clinical trials and vaccine efficacy studies, and will soon be applied to drug discovery and toxicology (Pulendran and Davis, 2020; Arunachalam et al., 2021; Roth et al., 2021; Zielinski et al., 2021).
